# Comparison of breathing pattern and diaphragmatic motion in patients with unilateral cervical radiculopathy and asymptomatic group

**DOI:** 10.1186/s12890-023-02804-x

**Published:** 2023-12-09

**Authors:** Raziyeh Yousefiyan, Amin Kordi Yoosefinejad, Reza Jalli, Iman Rezaei

**Affiliations:** 1 Student Research Committee, School of Rehabilitation Sciences, Shiraz University of Medical Sciences, Shiraz, Iran; 2 Physical Therapy Department, School of Rehabilitation Sciences, Shiraz University of Medical Sciences, 1 Abivardi Avenue, Chamran Blvd, P.O. Box: 71345-1733, Shiraz, Iran; 3 Rehabilitation Sciences Research Center, Shiraz University of Medical Sciences, Shiraz, Iran; 4 Medical Imaging Research Center, Shiraz University of Medical Sciences, Shiraz, Iran

**Keywords:** Respiration, Cervical radiculopathy, Diaphragm, Fluoroscopy, Assessment

## Abstract

**Background:**

The associations between neck pain and respiratory dysfunction were clarified in patients with neck pain. There is dearth of evidence on pulmonary dysfunction and diaphragmatic excursion in patients with unilateral cervical radiculopathy (CR). The purpose of this study was to compare the breathing pattern and diaphragmatic excursion in patients with unilateral CR with those in an asymptomatic group.

**Methods:**

Twenty-five patients with unilateral CR and 25 asymptomatic individuals aged between 30 and 55 participated in this study. Diaphragmatic motion, breathing pattern, active cervical range of motion and kinesiophobia were investigated in both groups by using fluoroscopy, manual assessment of respiratory motion (MARM), cervical range of motion device, and Tampa scale of kinesiophobia. Statistical significance was set at 0.05.

**Results:**

No statistically significant differences were found between the two groups with regard to sex, age and body mass index. The mean excursion of the hemi diaphragm on the involved side (the side of CR) was significantly lower than that on the uninvolved side in patients with unilateral CR with a large effect size. The excursion of the involved hemi diaphragm in patients was reduced compared to the matched hemi diaphragm in the control group. There was no significant difference between the hemi diaphragms excursion in the control group. The results of the MARM variables showed that the volume of breathing and the percentage rib cage motion in normal and deep breathing were significantly different between the two groups, but there was no significant difference in the balance of breathing between the two groups. Additionally, the active cervical range of motion was reduced in these patients in comparison to the control group, and it was less on the involved side than on the uninvolved side.

**Conclusion:**

The results of this study revealed a dysfunctional breathing pattern in normal and deep breathing and a unilateral reduction in diaphragmatic excursion on the side of radiculopathy in patients with unilateral CR compared to the control group.

## Introduction

Cervical radiculopathy (CR) is a common clinical disorder resulting from cervical nerve root entrapment or irritation secondary to spondylosis or disc herniation manifested as neck pain, sensory and motor deficits, or diminished reflexes. Symptoms may radiate into the dermatomal distribution of the affected nerve roots [1, 2]. Epidemiologic studies have shown that the annual incidence of CR is 83 per 100,000 with 95% of these patients have unilateral involvement [3].

Neck pain is a neuromusculoskeletal disorder that is accompanied by some dysfunctions in other parts of the body [4]. Previous researchers have found associations between the neck pain and respiratory dysfunction. The anatomical connection between the neck and thoracic region can lead to biomechanical changes in rib cage expansion and consequently, pulmonary function [5]. Decreased strength and endurance of neck muscles, decreased cervical range of motion (ROM), proprioception deficits, psychological dysfunctions, and other factors would contribute to respiratory dysfunctions in patients with chronic neck pain [6–9]. Perri et al. showed that 83% of patients with neck pain have faulty breathing patterns [10].

Respiratory dysfunction might be probable in patients with CR. A previous study demonstrated respiratory dysfunction in patients with CR as a decrease in spirometry pulmonary function parameters [11]. Involvement of phrenic nerve could be regarded as a contributing factor of respiratory dysfunction in these patients. The function of the diaphragm, as the primary inspiratory muscle providing nearly 70% of inspiratory air volume during quiet inspiration [12], might be impaired due to the involvement of common nerve roots with the phrenic nerve in patients with CR. O’Beirne et al. demonstrated that unilateral idiopathic diaphragmatic paralysis was associated with severe cervical spondylosis with radiculopathy [13]. In addition, there is a mechanical connection through the transversalis and thoracolumbar fascia between the cervical region and diaphragm. Due to this fascial bridge, dysfunctions of the diaphragm and neck muscles can mutually affect each other [14].

Chronic shortening and weakness of the diaphragm is associated with fiber loss, especially in the zone of apposition, leading to reduced force production and mechanical fault of the diaphragm [15, 16]. It may cause progressive compensatory use of other respiratory muscles, leading to chest or paradoxical breathing through changing the respiratory pattern and subsequent induced fatigue in the respiratory system, head and neck [17–19].

The diaphragm is subdivided into two hemi diaphragms, each innervated by the ipsilateral phrenic nerve [12]. We hypothesized that unilateral CR could lead to ipsilateral phrenic nerve and hemi diaphragm dysfunction, which appears as a weakness and excursion reduction of the ipsilateral hemi diaphragm. Asymmetric motion between the two hemi diaphragms followed by unilateral weakness, may lead to asymmetric rib cage expansion between the two sides of the body [20]. Patients with unilateral CR may intentionally or unconsciously refrain from performing breathing movements on the involved side to prevent symptom exacerbation.

Pain, together with its psychological, biological, and biochemical effects, reduced cervical ROM, fear of movement, and irrelevant neck postures could be regarded as other factors contributing to respiratory dysfunctions in patients with CR similar to those with chronic neck pain [5]. Due to the involvement of phrenic nerve roots and neurological symptoms, and sensory-motor deficits, respiratory dysfunction might be more prevalent in patients with CR. Unilateral CR and the consequent imbalance and asymmetric breathing pattern can make this even more complicated compared to patients with chronic neck pain.

The main aim of this study was to evaluate the respiratory pattern and hemi diaphragm excursion in patients with unilateral CR in comparison with the asymptomatic group.

## Method

### Participants

In this cross-sectional study, 25 patients with unilateral CR with an age range of 30 to 55 years and body mass index less than 30 (kg/m^2^) and 25 asymptomatic participants who were sex-, age-, body mass index-matched controls were conveniently recruited. Patients were included with unilateral C3-C7 CR and the involvement of C3-C4 or C4-C5 nerve roots as the necessary condition for at least 3 months, pain intensity between 3 and 10 based on numerical rating scale, and neck disability score between 15 and 50 based on neck disability index, considered moderate to severe pain and disability, respectively [21, 22]. All patients were clinically evaluated and diagnosed by a specialist confirmed by magnetic resonance imaging to determine the exact levels of compression.

Patients with chest or lung diseases, smoking habits, history of neck or thoracic surgeries, long-term use of steroid drugs, steroid injection in the last two weeks, musculoskeletal malformations such as scoliosis and kypho-scoliosis, acute respiratory infections, pregnancy, an annual X-ray dose of 50 milli Sieverts or more, professional athletes, physiotherapy of the neck region in the last month, use of nonsteroidal anti-inflammatory drugs in the last 24 h, respiratory complications of severe Coronavirus Disease 2019 or positive test in the last three months were excluded.

### Study setting

All participants were assessed at the Medical Imaging Research Center, Department of Radiology, Namazi Hospital and the laboratory of the Research Center of the School of Rehabilitation Sciences, Shiraz University of Medical Sciences, Shiraz, Iran. The study was performed between December 2022 and February 2023. All the participants signed an informed consent form before the commencement of the study. The study was approved by the local Ethics Committee of the Vice Chancellery of Research, Shiraz University of Medical Sciences (Ethics Number: IR.SUMS.REHAB.REC.1401.017).

### Study design

The study was designed to compare the respiratory function of the diaphragm and breathing pattern in patients with unilateral CR with those of asymptomatic control group. Respiratory function of the diaphragm was evaluated by fluoroscopy, and the pattern of breathing was examined with manual assessment of respiratory motion (MARM). Active cervical ROM was assessed using a cervical ROM device and fear of movement was assessed through the Tampa scale of kinesiophobia.

Sample size was calculated based on a pilot study. The difference of mean excursion of the hemi diaphragms between the involved and uninvolved sides during two predefined phases in patients with CR were considered as the primary outcomes of the study. Regarding an alpha level of 5% and a power of 80%, the minimum sample size was estimated to be 22 participants with an effect size of 0.80 and 28 participants, with an effect size of 0.71 during phase 1 defined as the difference between the hemi diaphragms excursion during inspiration and rest, and phase 2 determined as the difference between the hemi diaphragms excursion during inspiration and expiration, respectively.

### Procedures

#### Fluoroscopy assessment

Respiratory function of the diaphragm was evaluated using fluoroscopy (Toshiba tube, Japan, SN: A 50,106,536, Gen.num: 21,735) to measure the excursion of each hemi diaphragm during full expiration and inspiration. Fluoroscopy is a noninvasive and inexpensive imaging technique providing the recording of diaphragmatic movements in weight-bearing position as the most functional body position [23]. This is a reliable method for the evaluation of motion capable of producing real-time dynamic images and direct observation [24].

Based on the recommendation of the International Council on Radiation Protection, the annual permissible X-ray radiation dose is up to 50 milli Sieverts [25]. The estimated radiation dose is up to 2.6 milli Sieverts for fluoroscopic imaging of 7 to 10 min [16]. To minimize radiation exposure, fluoroscopic examinations were performed during three positions: normal breathing, deep inspiration and deep expiration in the posteroanterior view. The fluoroscopic images were viewed and saved on the Picture Archiving and Communication System software (www.infinitt.com).

The participants were asked to breathe deeply with maximal effort, including deep inhalation through the nose and deep exhalation through the mouth. Then, participants underwent fluoroscopic measurements of diaphragmatic movements in the standing position with the arms beside the body. They were asked to breath under the guidance of the radiologic specialist.

**Fig. 1:**
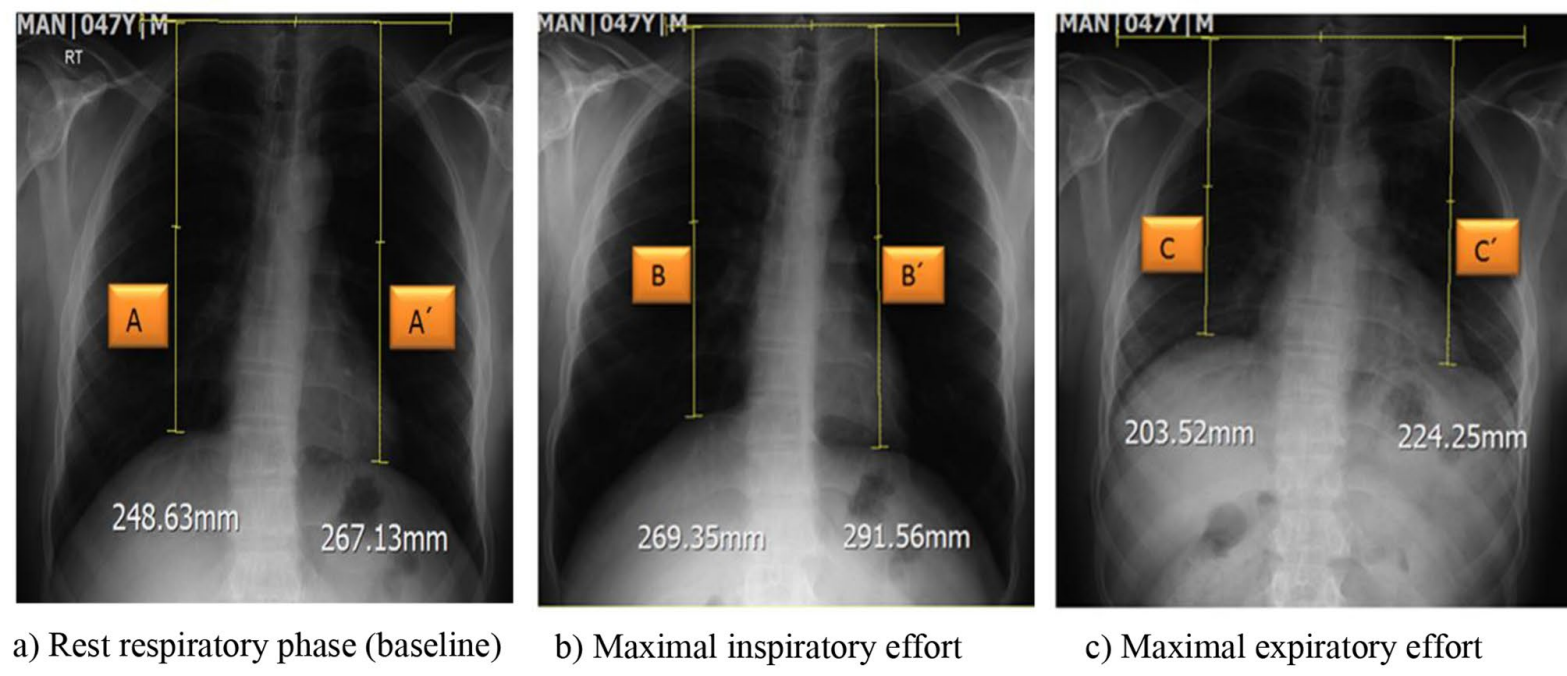
Fluoroscopic measurements in an asymptomatic 47 years old man. *Note*: A, B, C: vertical distance between the tubercle of the right first rib and dome of the right hemi diaphragm; A’, B’, C’: vertical distance between the tubercle of the left first rib and dome of the left hemi diaphragm

To measure the diaphragmatic motion, the vertical distance from the two landmarks including the dome of each hemi diaphragm and the tubercle of the first rib, was measured with the ruler tool in Picture Archiving and Communication System software. The excursion of the diaphragm in deep inspiration (phase 1) was determined by subtracting the vertical distance in deep inspiration from the obtained value in normal breathing. Moreover, the excursion of the diaphragm during deep inspiration and expiration (phase 2) was measured by calculating the difference in vertical distance between deep inspiration and deep expiration in millimeters [26, 27] (Fig. 1).

#### Manual assessment of respiratory motion

MARM is a clinical, time-efficient and inexpensive method of breathing pattern evaluation, with a very good interrater and intrarater reliability in patients with CR (unpublished manuscript/ under review) (interrater: intraclass correlation coefficient range = 0.71 to 0.82; intrarater: intraclass correlation coefficient range = 0.72 to 0.80), and a good inter- and intrarater reliability based on previous studies in asymptomatic people [28, 29]. The participants sat on a backless chair in a relaxed posture with their hands on knees. The examiner palpated the lower lateral rib cage with thumbs parallel to the spine and the ring and little fingers below the rib cage [28].

**Fig. 2:**
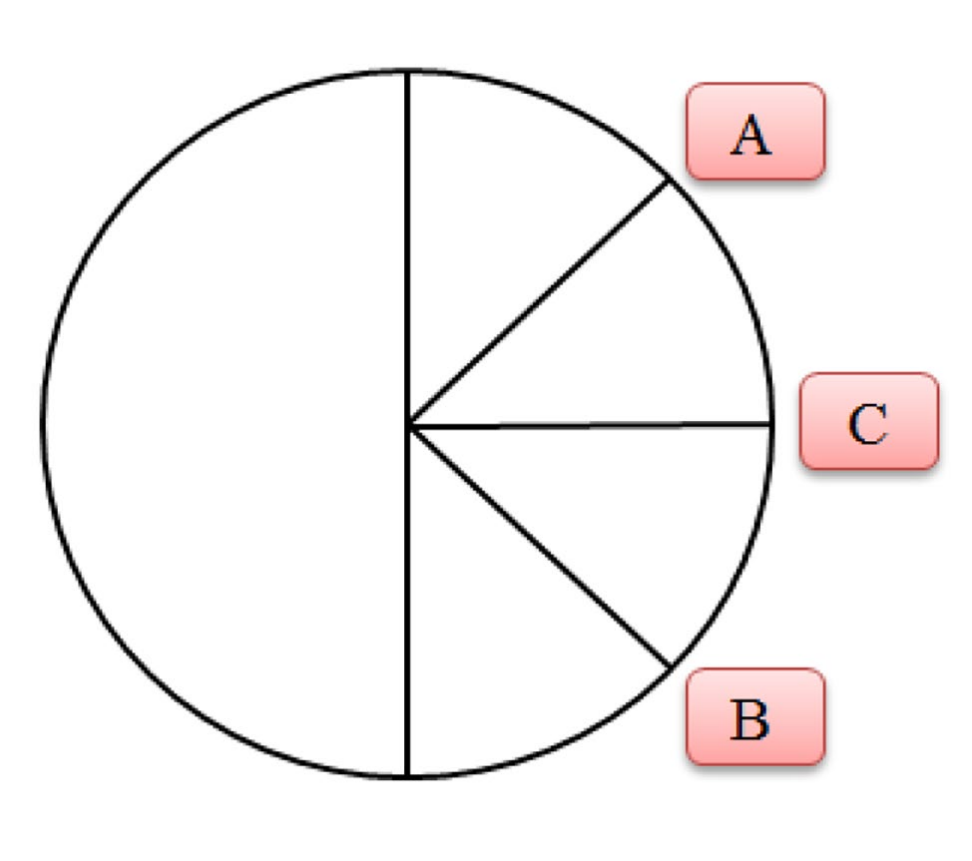
The manual assessment of respiratory motion graphic notation. *Note*: A: the vertical motion of the upper rib cage; B: the horizontal motion of the lower rib cage; C: horizontal line

Based on the overall sense of palpation, the examiner determined the dominancy of either the upper rib or lower rib cage and drew two lines in the pie chart of MARM. Line “A” represents the vertical motion of the upper rib cage, and line “B” represents the horizontal motion of the lower rib cage (Fig. 2). In normal and deep breathing, the respiratory pattern was recorded. If line “A” was farther from horizontal line (C), apical/upper chest breathing was predominant, while if line “B” was farther from “C”, abdominal/lower rib cage breathing was predominant [28]. Ideal breathing occurs when the respiratory effort is performed equally between the upper and lower parts of the chest. Three variables obtained from the MARM diagram are given below [28].

1. “Volume/area of breathing” is the sum of the thoracic and abdominal breathing volume, which is the angle between the upper line (A) and lower line (B). It is between 0 and 180 degrees. The larger the degree of AB, the greater is the respiratory volume.
2. “Balance of breathing” is the difference between AC and CB angles. This variable should be close to zero.
3. “Percent rib cage motion” is the angle above “C” line/ total angle (AB) ×100. It should be close to 50%.

#### Active cervical range of motion

A calibrated cervical ROM device (North Coast Medical, Inc. U.S.) was used to measure the active cervical ROM. This device was placed on the head. Three goniometers (two goniometers and one compass) were mounted on the device, and each was responsible for measuring the ROM in a motion plane. The participant sat comfortable on a chair with back support to avoid extra movements while they were looking forward. Each active cervical motion was repeated three times, and the average score was recorded for each motion [30, 31].

#### Tampa scale of kinesiophobia

The Tampa scale of kinesiophobia has 17 items assessing fear of movement and reinjury. Each question can be scored from 1 (completely disagree) to 4 (completely agree). Four questions (4, 8, 12 and 16) contain negative words. Its total score can range from 17 to 68, with higher scores indicating stronger avoidant fear beliefs. Scores greater than 37 indicate kinesiophobia. The scale was translated into Persian by Jafari et al. and validated by Askary-Ashtiani et al. in patients with neck pain, with acceptable reliability and validity [32, 33].

### Statistical analysis

The data were analyzed using Statistical Software SPSS (version 26.0; IBM Corp, Armonk, NY). Asymptomatic volunteers were matched on age, sex and body mass index. The normality of the data was confirmed by Kolmogorov‒Smirnov test. Regarding the normal distribution of data, a paired-sample *t*-test was used for within-group comparisons and an independent sample *t*-test was used for between-group comparisons. Statistical significance was set at 0.05 except for the excursion of hemi diaphragm in the radiculopathy group compared to the matched hemi diaphragm in the control group that was adjusted to *P*-value < 0.025 using Bonferroni correction. In addition, Cohen’s *d* was used to calculate the effects size of differences of variables between the two sides of the body in each group and differences between groups to clarify the clinical importance of the findings. According to the suggested values for effect size, a Cohen’s *d* of 0.2, 0.5, and 0.8 or greater are regarded as small, medium and large effects, respectively [34].

## Results

The study was conducted on 25 patients with unilateral CR (15 patients with right (Rt.) CR and 10 patients with left (Lt.) CR and 25 asymptomatic individuals. The fluoroscopic measures (the excursion of each hemi diaphragm), MARM items (volume of breathing, percent rib cage motion and balance), active cervical ROM and kinesiophobia were evaluated in each group. Table 1 shows demographic data of the participants in the groups.

**Table 1:** Demographic data and clinical characteristics of the groups

Variables	Radiculopathy [Mean (SD) or Ratio] (n = 25)	Control [Mean (SD) or Ratio] (n = 25)	*P* value
Age (years)	44 (7.42)	40.32 (7.91)	0.09
Sex (men/women)	7/18	9/16	0.53
Body mass index (kilograms/meter^2^)	25.1 (3.25)	24.96 (3.11)	0.95
Onset of pain (months)	8.40 (9.23)	N/A	N/A
Numerical rating scale (0–10)	6.68 (1.49)	N/A	N/A
Neck disability index (0–50)	20.40 (6.64)	N/A	N/A
Tampa scale of kinesiophobia (17–68)	41.28 (7.95)	N/A	N/A
Craniocervical angle (degree)	40.84 (6.35)	47.91 (7.42)	**0.001** ^*^
Thoracic kyphosis angle (degree)	28.39 (5.24)	23.91 (5.81)	**0.02** ^*^

Abbreviations: N/A, Not Applicable, ^*^: *P* < 0.05

### Outcomes

#### Fluoroscopic measures

Table 2 shows that the mean excursion of the hemi diaphragm was less on the involved side in patients with CR than on the uninvolved side in both the 1st (*P* < 0.001) and 2nd (*P* < 0.001) respiratory phases. However, there was no significant difference between the average excursion of the Rt. and Lt. hemi diaphragms in the 1st and 2nd respiratory phases in asymptomatic individuals.

**Table 2:** Excursion of hemi diaphragm (millimeter) in the radiculopathy and control groups during two respiratory phases

Variables	Radiculopathy [Mean (SD)]				Control [Mean (SD)]			
	**Involved side**	**Uninvolved side**	***P *** **value**	**ES**	**Right side**	**Left side**	***P *** **value**	**ES**
Phase 1 ^a^	16.27 (9.03)	24.56 (10.49)	**< 0.001** ^**^	0.84	30.77 (10.32)	31.24 (10.18)	0.29	0.04
Phase 2 ^b^	37.6 (15.76)	49.98 (18.70)	**< 0.001** ^**^	0.71	68.84 (21.13)	68.21 (21.33)	0.27	0.02

Abbreviations: ES, effect size, Note: a: the difference between the hemi diaphragms excursion during inspiration and rest; b: the difference between the hemi diaphragms excursion during inspiration and expiration

^**^: *P* < 0.001

The results of Table 3 show that the average excursion of the Rt. hemi diaphragm in the 1st (*P* < 0.001) and 2nd (*P* < 0.001) respiratory phases and the excursion of the Lt. hemi diaphragm in the 2nd respiratory phase (*P* = 0.002) were significantly less in the individuals with unilateral Rt. CR than the control group. The average excursion of Lt. hemi diaphragm in the 1st (*P* = 0.001) and 2nd (*P* = 0.001) phases of breathing was less in the individuals with unilateral Lt. CR than the control group.

**Table 3:** Excursion of hemi diaphragm (millimeter) in the radiculopathy group compared to the matched hemi diaphragm in the control group

Side of radiculopathy	Phase	Hemi diaphragm side	Radiculopathy [Mean (SD)]	Control [Mean (SD)]	*P* value	ES
Right cervical radiculopathy (n = 15)	1	Right	15.23 (9.73)	30.77 (10.32)	**< 0.001** ^**^	1.54
		Left	23.13 (11.96)	31.24 (10.18)	0.03	0.73
	2	Right	35.05 (15.80)	68.84 (21.13)	**< 0.001** ^**^	1.81
		Left	47.13 (17.15)	68.21 (21.33)	**0.002** ^*^	1.08
Left cervical radiculopathy (n = 10)	1	Right	26.71 (7.89)	30.77 (10.32)	0.27	1.02
		Left	17.82 (8.09)	31.24 (10.18)	**0.001** ^*^	1.45
	2	Right	54.25 (21.01)	68.84 (21.13)	0.07	0.69
		Left	40.09 (16.05)	68.21 (21.33)	**0.001** ^*^	1.48

Abbreviations: ES, effect size; Note: phase 1, the difference between the hemi diaphragms excursion during inspiration and rest; phase 2, the difference between the hemi diaphragms excursion during inspiration and expiration

^*^: *P* < 0.025, ^**^: *P* < 0.001

#### Manual assessment of respiratory motion values

Based on Table 4, significant differences were observed between the radiculopathy and control groups in volume/area of normal (*P* < 0.001) and deep breathing (*P* < 0.001) and percent rib cage motion in normal (*P* = 0.01) and deep breathing (*P* < 0.001)

**Table 4:** Manual assessment of respiratory motion variables in the radiculopathy and control groups

Variables	Radiculopathy [Mean (SD)]	Control [Mean (SD)]	*P* value	ES
Volume/area of NB (degree)	37.20 (7.88)	81.36 (8.61)	**< 0.001** ^**^	5.35
Balance of NB (degree)	-5.12 (7.91)	-3.44 (7.12)	0.43	1.13
Percent rib cage motion of NB (%)	43.02 (10.84)	47.74 (4.50)	**0.01** ^*^	0.56
Volume/area of DB (degree)	45.00 (8.29)	98.72 (13.6)	**< 0.001** ^**^	4.76
Balance of DB (degree)	14.08 (11.10)	10.00 (7.28)	0.13	0.43
Percent rib cage motion of DB (%)	66.19 (86.11)	54.79 (63.29)	**< 0.001** ^**^	0.15

Abbreviations: NB: normal breathing, DB: deep breathing. *: *P* < 0.05, **: *P* < 0.001

#### Active cervical range of motion

There were no significant differences in the mean flexion ROM between the two groups (*P* > 0.05), but the mean extension ROM was significantly less (*P* < 0.001) in the CR group than in the control group. The results regarding side bend and rotation ROM showed that the mean rotation (*P* = 0.002) and side bending (*P* < 0.001) to the involved side (the side of CR) were significantly less than those to the uninvolved side in the CR group. However, there were no significant differences in the mean rotation (*P* > 0.05) and side bending (*P* > 0.05) of Rt. and Lt. side compared to each other in the control group (Table 5).

**Table 5:** Flexion, extension, rotation and side bending ROM (degree) in the radiculopathy and control groups

**Variables**	**Radiculopathy [Mean (SD)]**				**Control [Mean (SD)]**		***P*** **value**	**ES**
Flexion	51.42 (10.46)				54.05 (11.17)		0.39	0.24
Extension	34.96 (10.64)				55.18 (10.97)		**< 0.001** ^**^	1.87
	**Involved side**	**Uninvolved side**	***P*** **value**	**ES**	**Right Side**	**Left side**	***P*** ** value**	**ES**
Side bending	29.54 (8.16)	36.69 (8.12)	**< 0.001** ^**^	0.87	43.42 (5.50)	42.90 (6.15)	0.37	0.08
Rotation	53.81 (6.32)	68.87 (18.54)	**0.002** ^*^	0.92	74.33 (8.63)	73.86 (8.78)	0.41	0.05

Abbreviation: ES, effect size. ^*^: *P* < 0.05, ^**^: *P* < 0.001

## Discussion

This study was conducted to compare the breathing pattern and diaphragmatic excursion in patients with unilateral CR with asymptomatic group. Our results confirmed a dysfunctional breathing pattern and unilateral reduction of diaphragmatic excursion on the side of radiculopathy in these patients with a moderate to large clinical significance. The excursion of the involved hemi diaphragm in CR patients with CR was significantly less than the intact hemi diaphragm (Table 2) and matched hemi diaphragm in asymptomatic group (Table 3). The results are confirmed clinically with a large effect size on the excursion restriction of the involved hemi diaphragm.

It can be summarized that one of the causes of diaphragmatic dysfunction in patients with unilateral CR, was motion restriction of the diaphragm secondary to the involvement of common irritated nerve roots with the phrenic nerve. Prolonged compression of the ipsilateral phrenic nerve roots can eventually lead to trophic changes and disturbance in nutrition and function of the nerve, and consequently diaphragmatic weakness and altered breathing pattern [12, 35].

Other potential causes of respiratory dysfunction are mild damage to respiratory control tracts following chronic compressive stress or potential compensatory reactions of cervical spinal cord [36]. The spinal cord is a vital organ containing descending pathways in the dorsolateral columns, which are essential pathways for respiratory muscle activity [37]. Lesions in the cervical spinal cord, such as compression of the cervical nerve roots, can lead to respiratory disorders by disrupting the descending pathways [36].

In line with our results, Fahad et al. have shown the existence of respiratory dysfunction and a decrease in spirometry parameters in patients with spinal canal stenosis with CR due to partial damage of the phrenic nerve [11]. In addition, according to Kang et al., diaphragm weakness could be the consequence of cervical C_3_ and C_4_ root radiculopathy [35]. The results of the study by Bhagavatula et al. have shown that the decrease in respiratory parameters, especially forced vital capacity, in patients with chronic pressure on the cervical roots above C_5_ is due to diaphragmatic dysfunction [38].

Another pathomechanism of this respiratory dysfunction can be the decrease of intercostal muscles tone and imbalance of the autonomic nervous system, which was arisen in chronic compression of cervical nerve roots. Predominant sympathetic activity, instead of parasympathetic neurons in normal breathing, may lead to dysfunctional breathing [36–38].

Regarding the MARM variables, we found significant differences in the volume of breathing and percent rib cage motion between the groups in both respiratory phases; but, there was no significant difference in the balance of breathing between the groups. In the radiculopathy group, abdominal breathing was more dominant in normal breathing, and chest breathing was predominant in deep breathing. However, the control group had a more balanced breathing pattern. With regard to the values of Cohen’s *d* in MARM variables between the groups, CR showed a clinical large effect on volume of breathing in both respiratory phases (Table 4).

Previous studies are in line with us and have confirmed the dysfunctional breathing pattern in 83% of people with neck pain [10]. In the study by Kapreli et al., decreased respiratory capacity and weakness of respiratory muscles were reported in people with chronic neck pain [7]. Perri et al. found a link between chest breathing pattern and history of chronic cervical pain [10].

Altered breathing pattern leads to decreased lung volume, hypoxia, respiratory alkalosis, and increased the central and peripheral excitability of the nervous system; this negative cycle can again lead to a disturbance in the respiratory pattern. Additionally, chest breathing patterns can cause excessive activity and fatigue of superficial neck flexor muscles, such as the sternocleidomastoid [39].

Regarding the cervical ROM, the range of extension was statistically and clinically less in the patients with CR compared to the control group (Table 5). However, there was no significant difference in flexion ROM between the groups. Rotation and side bending to the involved side in the CR group were significantly less than those to the uninvolved side, clinically confirmed with large effect size (Table 5). Several studies have shown a decrease in cervical ROM in all planes in patients with chronic neck pain and CR [40–43]. In a systematic review by Kahlae et al., cervical ROM was significantly correlated to the respiratory parameters [41].

It seems that decreased cervical ROM could be due to neck pain and fear of movement. In chronic neck pain, weakness of deep neck flexor and extensor muscles lead to over activity of superficial muscles and results in fatigue and altered length-tension relationship of these muscles. Dysfunction of the superficial muscles of neck, as the respiratory accessory muscles, may lead to breathing pattern dysfunction [5].

Decreased cervical ROM can augment respiratory dysfunction by limiting the myofascial mobility [5]. Due to the anatomical (musculo-skeletal) connection of the cervical spine with the chest region, reduced neck movements can negatively affect the mechanics of the chest and reduce chest expansion, which would lead to the respiratory dysfunction [5, 41].

Regarding the craniocervical and thoracic kyphosis angle in our patients, patients with CR had forward head posture (FHP) and hyper-kyphosis. There are inconsistent findings regarding the relationship between FHP and neck pain. Some of the findings confirmed the relationship between these two parameters [44, 45]; however, based on a recent study, there were neither difference in FHP between patients with neck pain and asymptomatic people nor correlation with neck pain [41].

In addition, there are various findings on the impact of FHP on the respiratory function. Some studies have reported that FHP is a common postural malalignment in patients with chronic neck pain with a negative impact on respiration [46–49]. In contrast, Dimitriadis et al. showed that patients with chronic neck pain did not differ from the control group in terms of FHP, despite proven respiratory dysfunction [40]. Additionally, Ahmed et al., compared the excursion of the diaphragm in patients with nonspecific chronic neck pain in two groups with FHP and normal head posture. The results showed decreased diaphragmatic excursion in patients with nonspecific chronic neck pain with FHP, but the difference was not statistically significant [50].

There is an interesting explanation that in patients with neck pain, breathing dysfunction improve by increased FHP. Therefore, FHP in these patients does not seem to be solely a maladaptive posture, but a compensatory mechanism to increase the airflow [7, 10, 51]. The results of our study agree with those by Patwardhan et al., who reported increased FHP in these patients [52]; however, the results of Lopez et al. and Ghamkhar et al., are contrary to the results of the present study. They showed that the craniocervical angle did not differ between patients with chronic neck pain and control group [42, 53].

FHP may be considered a compensatory mechanism to reduce cervical nerve root compression in our study [52]. Increased upper thoracic kyphosis and radicular symptoms in patients with CR are compensated by increased craniocervical angle. Therefore, the neural foraminal space of the lower cervical spine is increased due to FHP and reduced nerve root compression [52].

Patients with CR showed relatively high levels of fear of movement (41.28 ± 7.95 of 68). In a study by Dimitriadis et al., respiratory dysfunction and significant decrease in respiratory parameters in patients with chronic neck pain, were associated with psychological factors, including fear of movement [54]. Additionally, Woods et al., reported fear of movement in patients with CR [43].

Based on the previous studies, there is a respiratory dysfunction in people with chronic neck pain and CR. Fear of movement, as a psychological parameter, can aggravate the dysfunctional breathing by affecting the blood chemistry. Kinesiophobia is the second main reason for the reduction of carbon dioxide gas pressure in the blood, due to the increase of secretion of adrenaline; hence, affects ventilation. Therefore, breathing pattern will be changed and lead to dysfunctional breathing [5, 39].

Based on our findings, diaphragmatic excursion and breathing patterns changed in patients with unilateral CR. Despite its importance, breathing assessment is often ignored in clinical environments. It is suggested that breathing assessment be added to physical examinations in patients with CR.

One of the limitations of this study was that the direct comparison of fluoroscopy and MARM variables was not possible due to different positions of examination (standing versus sitting). Clinical trial studies are warranted to prescribe a breathing exercise program for patients with unilateral CR and to evaluate the improvement of respiratory patterns. We suggest that future studies be performed to investigate the effects of routine physiotherapy on respiratory function in patients with CR.

## Conclusion

The results of this study showed diaphragmatic dysfunction and altered breathing patterns in patients with unilateral CR. Our findings revealed unilateral excursion reduction of the diaphragm on the side of the CR and a faulty breathing pattern as chest breathing during deep inspiration. Based on the findings of this study, it could be suggested to adjunct respiratory assessments to the physical examinations of these patients in clinical environments.

## Data Availability

The datasets generated and analyzed during the current study are available from the corresponding author on reasonable request.

## Abbreviations

CR: Cervical Radiculopathy
MARM: Manual Assessment of Respiratory Motion
ROM: Range of Motion
FHP: Forward Head Posture
Rt.: Right
Lt.: Left
